# Co-solvent polarity tuned thermochromic nanotubes of cyclic dipeptide–polydiacetylene supramolecular system†

**DOI:** 10.1039/d0ra05656a

**Published:** 2020-09-24

**Authors:** Mohammed Iqbal Khazi, Chenikkayala Balachandra, Geon Shin, Gang-Hee Jang, Thimmaiah Govindaraju, Jong-Man Kim

**Affiliations:** Institute of Nano Science and Technology, Hanyang University Seoul 04763 Korea jmk@hanyang.ac.kr; Bioorganic Chemistry Laboratory, New Chemistry Unit, School of Advanced Materials (SAMat), Jawaharlal Nehru Centre for Advanced Scientific Research Jakkur P.O. Bengaluru Karnataka 560064 India tgraju@jncasr.ac.in; Department of Chemical Engineering, Hanyang University Seoul 04763 Korea

## Abstract

The cooperative non-covalent interactions arising from structurally integrated multiple molecules have emerged as a powerful tool for the creation of functional supramolecular structures. Herein, we constructed cyclic dipeptide (CDP)–polydiacetylene (PDA) conjugate (CDP–DA) by introducing cyclo(l-Phe-l-Lys) to the linear 10,12-pentacosadiynoic acid. Owing to extensive hydrogen bonding characteristics, together with structural chirality of cyclo(l-Phe-l-Lys) and strong π–π stacking diacetylenic template, CDP–DA generated supramolecular nanotubes. The structural visualization using scanning and transmission electron microscopy revealed chloroform/methanol co-solvent polarity tuned morphological transformation of intrinsic lamellar assemblies into nanotubes comprising single-wall and multi-wall structure. The mechanistic understanding by X-ray diffraction patterns confirms bilayer organization in lamellar structure, which forms nanotubes *via* a gradual lamellar curling-to-scrolling process. The supramolecular CDP–DA nanotubes are transformed into the rigid covalently cross-linked blue-phase polydiacetylene (CDP–PDA) by UV irradiation. Very interestingly, the blue-phase nanotubes display reversible thermochromic changing temperature up to 150 °C with excellent repeatability over a dozen thermal cycles. This work provides an efficient strategy for precise morphological control and aiding the perspective for development in nanostructures for functional devices.

## Abstract 2

Co-solvent controlled fabrication of thermo-responsive chromogenic nanotubes of a cyclic dipeptide–polydiacetylene supramolecular system.

## Graphical abstract

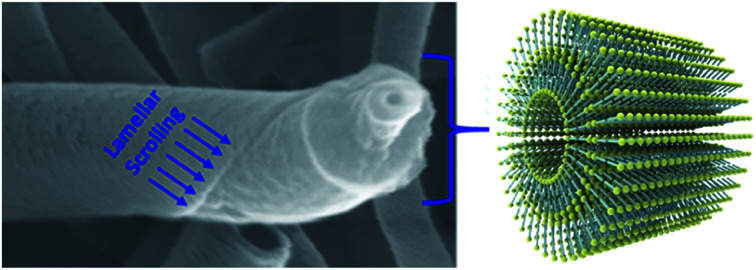

## Introduction

The propensity of self-assembly extended to create hierarchical supramolecular polymer architectures has attracted increasing interest recently.^1–6^ Self-assembled polymer architecture reflects the integrated structural and functional features of monomer components. The self-assembly mechanism is an autonomous molecular organization with high geometric precision and it is strongly governed by the cooperative multiple non-covalent interactions.^7–10^ These interactions serve as a binding force across multiple self-assembling subunits and ensure the degree of sequence-specificity to generate stable supramolecules. More importantly, the reversible and tunable nature of non-covalent interactions endows these supramolecules with intriguing properties of stimuli-responsiveness and reversibility. The judicious modulation of the molecular framework affords dynamic architecture that can generate an assembly-related functional response.^11–16^ Particularly, controlling the hydrophobic/hydrophilic balance, nature of the connectivity and structure of the hydrophobic/hydrophilic components in molecule provides new opportunities for designing smart materials for advanced application in biomedical and nanotechnology.^17–27^

Polydiacetylenes (PDAs) are intrinsically supramolecular polymers generated *via* hierarchical self-assembly of monomeric diacetylene (DAs).^28–32^ Importantly, these self-assembled DAs can integrate into robust covalently linked PDAs by facile UV-induced topochemical polymerization. PDAs are typically chromophoric due to the extended π-conjugated network and absorb visible light. Owing to the non-covalent interactions promoted self-assembled structure, PDAs exhibit colorimetric (typically blue-to-red) and optical transitions in response to external stimuli, and these unique properties of PDAs have been extensively applied for sensing applications.^33–44^ In addition, a wide array of structural morphologies of PDAs can be constructed by the precise introduction of head groups to DAs.^45–50^

Cyclic dipeptides (CDPs) are the simple cyclic peptide scaffolds with a rigid 2,5-diketopiperazine (DKP) as a basic skeleton, and generally explored as a versatile scaffolding component in the field of supramolecular chemistry.^51–57^ The *cis*-amide functionality of CDPs induces sequential organization of adjacent molecules *via* hydrogen bonding (N–H⋯O) as well as other non-covalent interactions, thus leading to the generation of higher-ordered artificial functional superstructures. The rich structural diversity of amino acids and with recent advances in the synthetic strategies utilizing combinatorial chemistry, an extended library of symmetric and asymmetric CDPs are easily accessible.^58,59^ CDP based supramolecular architectures are widely explored for a variety of applications ranging from therapeutics to materials.^60–68^

Supramolecular assemblies of linear monomers offer a facile protocol for the creation of polymer architecture of various morphologies, however, the construction of covalently connected nanotubes is challenging and often limited to certain molecular structure and polymerization techniques. The reason for this limitation is that nanotube formation generally requires a higher-ordered molecular packing and definite directionality. In order to induce favorable molecular organization for nanotube formation, amphiphiles consist of hydrophobic and hydrophilic components are often utilized. Previously we reported chromogenic nanotubes generated from CDP–PDA conjugates prepared by coupling cyclo(-Gly-Ser) and *cis*-cyclo(-Ser-Ser) with 10,12-pentacosadiynoic acid (PCDA).^69^ To further explore the design diversity and structural influence of CDP on morphology and functional property of integrated CDP–PDA system, herein we constructed a new CDP–PDA derivative by introducing cyclo(l-Phe-l-Lys) to the PCDA *via* an amide linkage. The results of this study show co-solvent polarity tuned morphology transformation from lamellar structure to nanotubes *via* scrolling mechanism. The morphological features were studied by scanning and transmission electron microscopy, and X-ray diffraction analysis. The CDP–DA supramolecular nanotubes undergo UV-induced topochemical polymerization, leading to blue-phase covalently crossed-linked PDA nanotubes. Most importantly, the blue-phase CDP–PDA nanotubes generated a brilliant eye-sensitive reversible blue-red-blue colorimetric sensory response to heat for up to 150 °C. The tendency for topochemical polymerization and thermo-responsive colorimetric responses was observed by spectral analysis.

## Experimental

### Synthesis

Synthesis and characterization of CDP–DA is described in the ESI.†

### Specimen preparation

Fabrication of supramolecular assemblies CDP–DA (3 mg) was dissolved in 300 μl of solvent by means of sonication and heating (∼55 °C) to clarity. The resulting clear solution was cooled in the refrigerator (−7 °C) for self-assembly and then drop-casted on a slide glass. The specimen was dried at room temperature.

### Thermochromic sensing

The monomeric CDP–DA was transformed into a blue-phase PDA by irradiating with 254 nm UV light (2 mW cm^−1^) for 5 min. The blue-colored test slides were heated on a hot plate at the rate of 10 °C per min and monitored for the colorimetric response.

## Results and discussion

### Design and synthesis of CDP–DA supramolecule

**Fig. 1:**
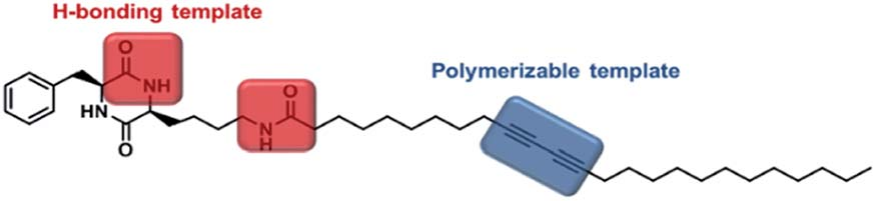
Molecular structure of cyclic dipeptide–diacetylene conjugate (CDP–DA).

**Fig. 2:**
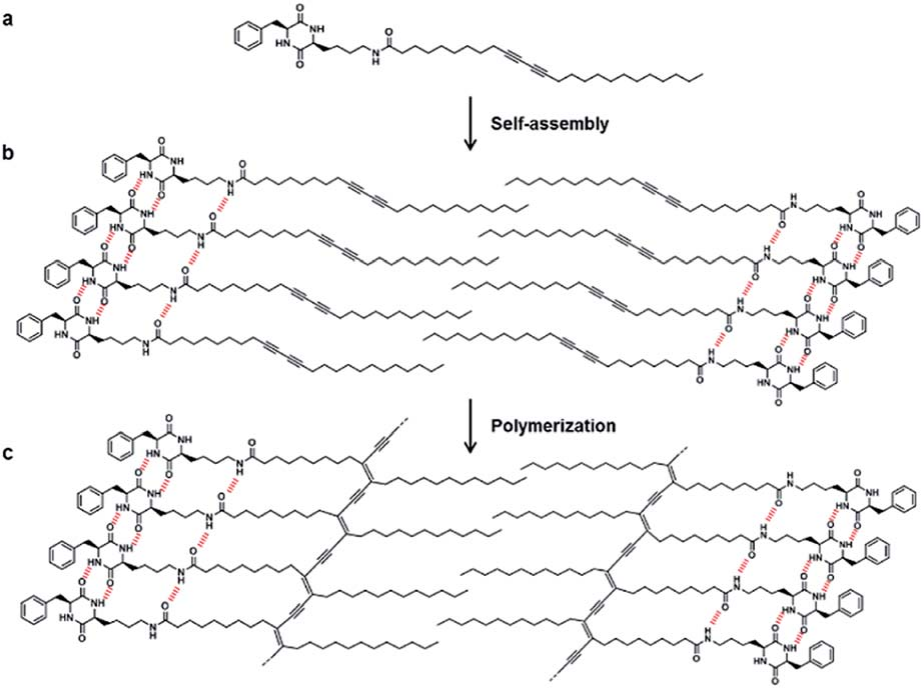
Illustration of the self-assembled CDP–DA *via* hydrogen bond interactions and UV-induce PDA formation. (a) Structure of CDP-DA. (b) Self-assembled CDP–DA. (c) Formation of polymerized CDP–PDA upon UV irradiation.

The strategy for the fabrication of the covalently linked chromogenic nanotubes was achieved by coupling the chiral hydrogen-bonding motif, CDP, with a photopolymerizable DA template. As seen in Fig. 1, the CDP–DA represents a typical amphiphilic molecular frame in which a semi-rigid cyclo(l-Phe-l-Lys) is connected to the linear 10,12-pentacosadiynoic acid (PCDA) chain *via* an amide linkage in a head-to-tail fashion. The *cis*-amide functionality in cyclo(l-Phe-l-Lys) with 2H-bond acceptor and 2H-bond donor sites forms sequential intermolecular amide–amide (N–H⋯O) hydrogen bonds between adjacent molecules, and thus enable 2,5-diketopiperazine core to take up-regulated molecular structures with a high degree of order. The π–π stacking characteristic of the diacetylene template and complementary hydrogen bonding interactions of the amide linkage directs the PCDA tail to adopt optimal orientation for topochemical polymerization. A clear illustration from Fig. 2 indicates that the initial step of the CDP–DA self-assembly is the formation of a sequential H-bonded network *via* a cooperative mechanism of non-covalent hydrogen bonding interactions and π–π stacking. The chiral and conformationally constrained CDP head group serves as powerful inducers, which restrict the local accessible structural conformations and induces helicity in the supramolecular association to generate the tubular structure.

For the synthesis of CDP–DA, first, an asymmetric DKP-scaffold, cyclo(l-Phe-l-Lys) was synthesized by following the previously reported procedures.^70^ The CDP finally coupled with PCDA *via* an amide bond (see the ESI† for the scheme and experimental details). The structures of the synthesized products were confirmed by using IR, ^1^H, ^13^C NMR, and MALDI-TOF spectral methods (see Fig. S1 and S2 in ESI†). The precisely positioned chiral center of the head group induces the helical aggregation process to generate nanotubular structures. In the subsequent step, these supramolecular nanotubes are expected to undergo UV promoted topochemical polymerization to form chromogenic π-functional superstructures.

### Fabrication and morphological characteristics of the self-assemblies

Self-assembly study of CDP–DA was carried out in different solvents such as chloroform, dichloromethane, ethanol, tetrahydrofuran, and in binary chloroform/methanol mixes at varying volume ratios (Table S1†). The self-assembled nanostructures are fabricated by employing a simple drop-casting technique on a glass substrate.

**Fig. 3:**
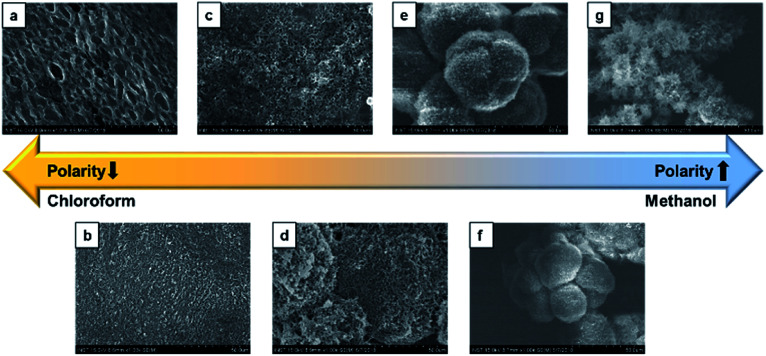
SEM images of the different morphologies of CDP–DA at varying chloroform : methanol polarity ratio. (a) Chloroform, (b) 3 : 1, (c) 2 : 1, (d) 1 : 1, (e) 1 : 2, (f) 1 : 3, (g) methanol.

**Fig. 4:**
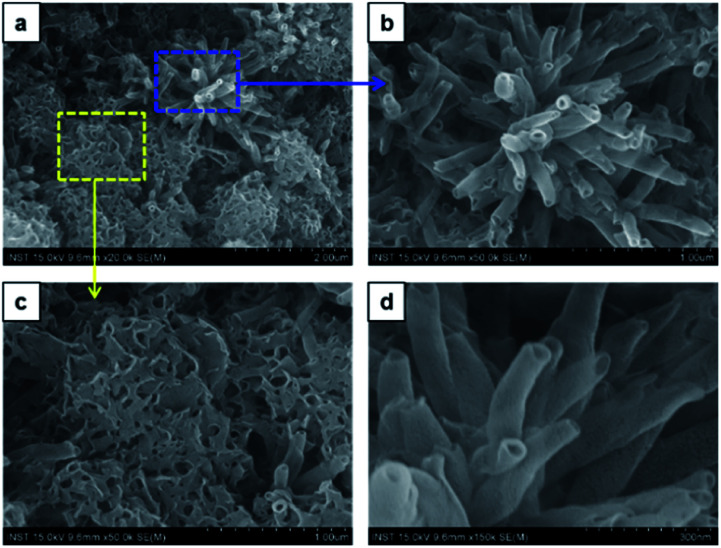
SEM images of the two co-existing morphologies of CDP–DA in chloroform : methanol mixture at 1 : 1 v/v ratio (a) dandelion-like microsphere structures generated. (b) Magnified image of the blue marked region in panel-a. Hollow nano tubular structures. (c) Magnified image of the yellow-marked region in panel-a. Sheet-like structure. (d) Magnified image of the panel-b.

To investigate the solvent assisted self-assembled morphological diversity of CDP–DA and to access the information on structural features, scanning electron microscopy (SEM) and transmission electron microscopic imaging (TEM) was performed. Furthermore, X-ray diffraction analysis (XRD) was used to determine the self-assembly behavior and packing pattern of supramolecular structures. Examining SEM images reveals the formation of a variety of nanostructures for the self-assembled aggregates of CDP–DA (Fig. S3 in ESI†). An interesting co-solvent polarity tuned morphology transformation behavior was observed for CDP–DA in a chloroform/methanol solvent system. As seen in Fig. 3, multiple self-assembled structures consisting of fibrous xerogel to perfect tubular morphology evolved for CDP–DA at the varying ratio of chloroform/methanol mixes facilely by drop-casting on a glass substrate. In less polar chloroform, CDP–DA assembled into xerogel having a porous network-like external structure. The addition of polar methanol to the chloroform solution in the ratio of 3 : 1 and 2 : 1 (v/v chloroform : methanol) modulated the morphology of aggregates into intertwined nano-fibrous gel network. In both samples, the nanofiber structures with the high aspect ratio are observed. Interestingly, at an equivalent ratio of chloroform–methanol, 1 : 1 v/v, a sharp gel-to-solid transition was observed. Examining the SEM image shown in Fig. 4 revealed a new morphology having dandelion-like micron-scale spheres. Upon closer examination of the SEM image displayed in Fig. 4a, microspheres were observed to contain two different kinds of coexisting structures (regions separately marked with blue and yellow colors). As seen in the magnified images of marked regions displayed in Fig. 4b and c, dandelion-like microsphere consists of clearly identifiable wrinkled sheet-like (lamellar sheet-like arrangement) and tubular structure.

**Fig. 5:**
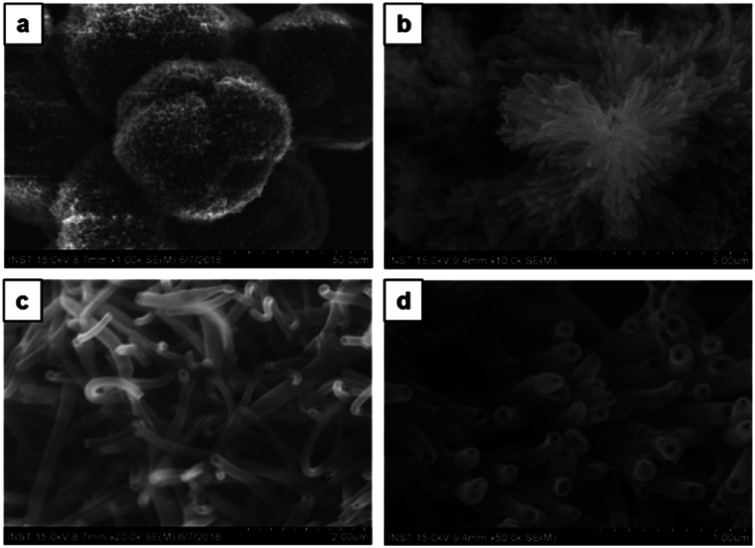
SEM images of the CDP–DA in chloroform : methanol mixture at 1 : 2 v/v ratio (a) dandelion-like microsphere structures associated as bunch. (b) Magnified image of the panel-a. Microspheres composed of densely packed nanotubes. (c and d) Magnified image of the panel-b.

Next, upon changing the volume ratio of chloroform/methanol by the incremental addition of methanol to chloroform solution in the ratio 1 : 2 v/v (chloroform : methanol), CDP–DA self-assembled into microspheres with densely packed tubular structures (Fig. 5). These hierarchical microspheres' structures can be seen associated as a bunch. The higher ratio of methanol believes to increase the stability of the assemblies in solution by controlling the non-covalent interactions and assist in the uniform tuning of the morphology into nanotubes. The image displayed in Fig. 5c reveal the tubes with ‘open mouth’ ends of a nanometric diameter. In order to get further insight into the morphological evolution of nanotubes, magnified SEM visualizing was performed. As seen in Fig. 5d, the nanotubes are formed of single-wall and multi-wall structures, which indicate the rolling-up process of lamellar sheet-like structure into tubes. The consistent formation of long and more perfect tubular morphology of microsphere confirms that the addition of polar methanol in a two-fold increment to chloroform solution of CDP–DA (1 : 2 v/v) is appropriate ratio added to stabilize aggregates in process of supramolecular self-assembly.

**Fig. 6:**
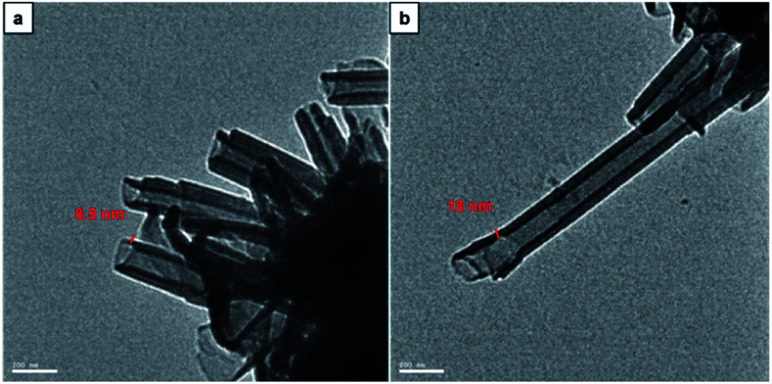
TEM images of CDP–PDA nanotubes generated in mixed chloroform and methanol solution at the ratio of 1 : 2 (v/v). Nanotube thickness (a) 8.5 nm and (b) 18 nm

Morphology analysis by imaging with TEM was performed to further confirm the tubular characteristic and to determine the structural dimensions of CDP–DA assemblies. The corresponding TEM micrographs are shown in Fig. 6. As can be seen, nanotubes with a hollow interior of nanometric diameter are visible. Careful assessment reveals the varying thickness of the tubular wall, which has a range with a minimum of 8.5 nm to a higher value of 18 nm. The powder X-ray diffraction study of CDP–DA provides a clear insight into the self-assembly behavior and possible packing structure of nanotube formation from intrinsic lamellar structures. The X-ray diffraction patterns of pure nanotube structure showed six peaks at 2*θ* = 2.12, 4.36, 6.56, 8.76, 10.82, 13.2 (Fig. S4 in ESI†). The interlamellar distance was calculated using Bragg's law, which was found to be 4.06 nm. With the length of CDP–DA being 4 nm, a bilayer lamellar self-assembly of the molecules can be proposed, which intern formed into a tubular structure. Notably, the calculated length is in agreement with the thickness of the tubular wall observed in TEM images.

**Fig. 7:**
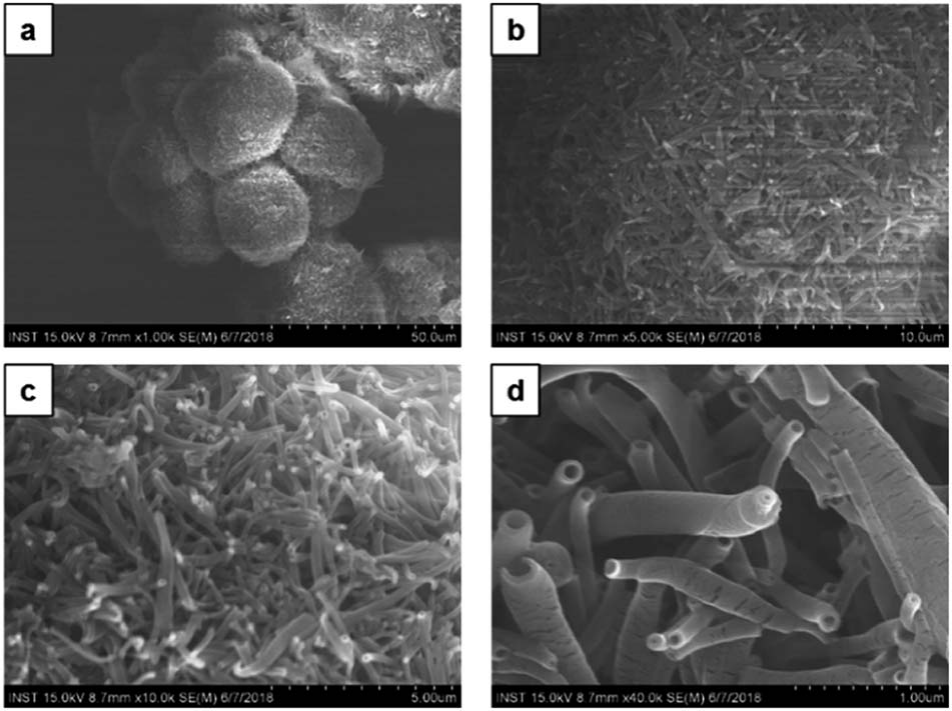
SEM images of the CDP–DA in chloroform : methanol mixture at 1 : 3 v/v ratio (a) dandelion-like aggregates composed of nanotubes. (b) Magnified image of the panel-a. Aggregates composed of densely packed nanotubes and curled sheets. (c and d) Magnified image of the panel-b.

**Fig. 8:**
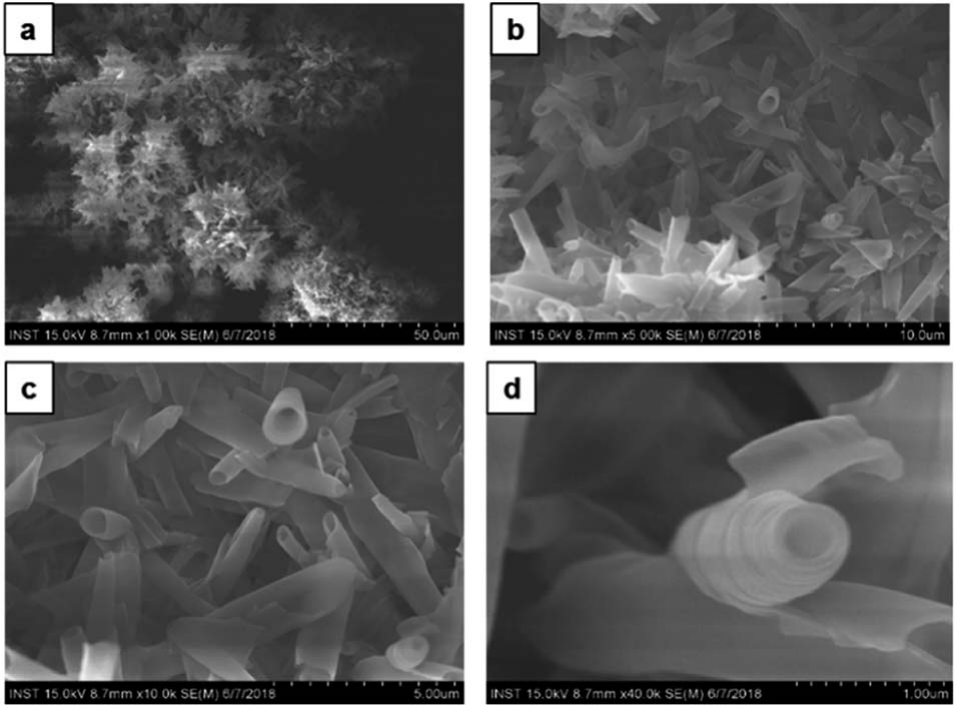
SEM images of the CDP–DA in pure methanol (a) flower-like aggregates composed of nanotubes. (b) Magnified image of the panel-a. Aggregates composed of densely packed nanotubes and bend/curved sheets. (c and d) Magnified image of the panel-b.

Furthermore, by increasing the methanol content in chloroform solution in a ratio of 1 : 3 v/v (chloroform : methanol) no morphology change was observed. A similar type of dandelion-like microsphere morphology with densely intertwined nanotubes formed, which has open-end single-wall and multi-wall structures (Fig. 7). Finally, the self-assembly of CDP–DA in highly polar methanol displays a self-assembled flower-like aggregate with inconsistent tubular structures (Fig. 8a). Close inspection of SEM images shows that the tubes are loosely packed and relatively shorter in length than that obtained in chloroform/methanol solvent mixture in ratios at v/v 1 : 2 and 1 : 3 (Fig. 8b and c). Moreover, it predominately consists of the curved and bends plate-like structures (Fig. 8d), which underlines again the sensitivity of the polarity of the solvent system for favourable non-covalent interactions. These morphological observations suggest that chloroform/methanol ratio of 1 : 2 v/v is a good co-solvent system to produce uniform nanotubular morphology with consistently high quality.

**Fig. 9:**
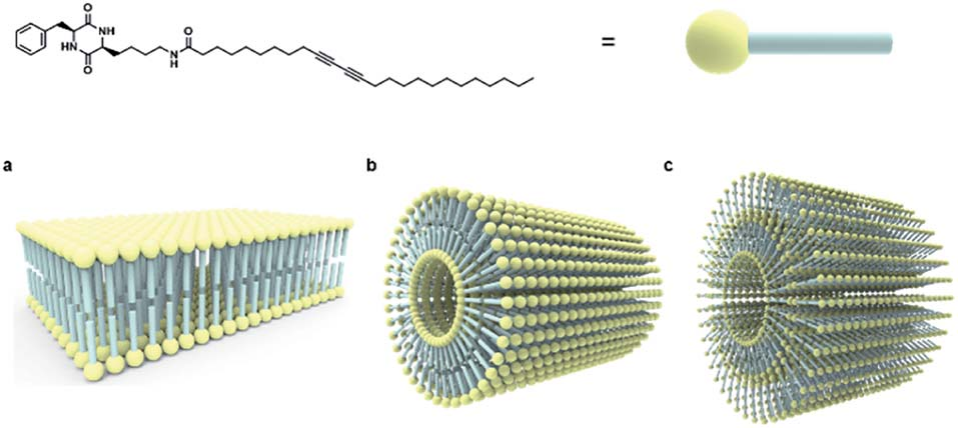
Schematic representation of self-assembly process of CDP–DA nanotubes. (a) Bilayer assembly of CDP–DA. (b) Single-wall structure. (c) Multi-wall structure.

Based on collective information on structural characteristics and theoretical calculation, it could be inferred that the formation of CDP–DA nanotubes assumes a polarity-driven two-step self-assembly process combining lamellar packing and scrolling-behavior. A schematic model is proposed for the formation of nanotubes from CDP–DA bilayer assemblies (Fig. 9). First, the CDP–DA adopts a bilayer structure orientated in a tail-to-tail interface and concurrently assemble into a lamellar sheet-like structure before assembly into nanotubes. The extensive hydrogen bonding interaction arising from CDP moieties and bridging amide functionality, and π–π stacking characteristics of the diacetylene template serve as a stitching force to organize CDP–DA into highly ordered lamellar sheets (Fig. 9a). Next, the lamellar sheets start to curl when the ratio of methanol to chloroform increased in a two-fold amount (1 : 2 v/v chloroform/methanol). These curled/curved sheets continue to roll up and finally assembled into the hollow nanotubular structure. Considering the dimensions of the tubular wall from microscopic analysis and packing pattern from XRD study, and the theoretically predicted bilayer length, two possible scrolling pathways for the generation of single-wall structure and multi-wall structure can be proposed (Fig. 9b and c).

### Covalently linked PDA nanotubes

**Fig. 10:**
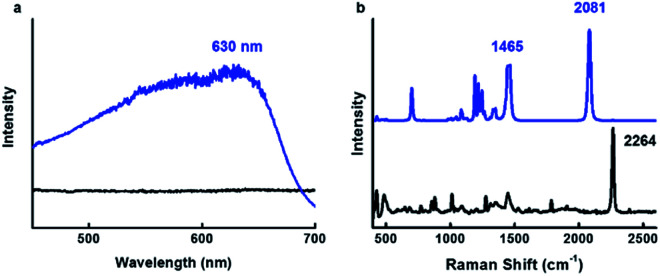
Topochemical polymerization of CDP–DA in chloroform : methanol mixture at 1 : 2 v/v ratio. (a) UV-vis (b) Raman spectra of CDP–DA before (black line) and after (blue line) 254 nm UV irradiation.

After identifying the ideal co-solvent polarity to induce favorable non-covalent interactions for the consistent production supramolecular CDP–DA nanotubes, and characterization of tubular morphology, the topochemical polymerization was attempted to integrate supramolecular CDP–DA nanotubes into covalently cross-linked rigid polymeric nanotubes (CDP–DA) by facile UV irradiation (UV 254 nm, 2 mW cm^−2^). Upon exposure to UV light, the white color CDP–DA solid turns to typical blue color because of polymerization. The resulting material was characterized by reflection-absorption spectroscopy and Raman spectroscopy. The absorption peak at 630 nm appeared on the UV-vis spectra assign the formation of the conjugated ene–yne PDA chain (Fig. 10a). Further insight on the PDA formation was confirmed by using Raman spectroscopic analysis (Fig. 10b). The two distinct bands associated with the conjugated ene–yne template appeared at 1465 (C=C) and 2081 (C≡C) cm^−1^ respectively, while, the yne–yne band at 2264 cm^−1^ (C≡C) for monomer DA disappeared. These results indicate that the presence of intermolecular non-covalent interactions facilitated the formation of hierarchical self-assemblies, which is adequate to provide an optimal geometry for the DA monomers to undergo the efficient photopolymerization. Since the PDA has critical solubility issues, it was difficult to determine the molecular weight of the polymer by GPC analysis. However, the theoretical calculation for the linear PDA to absorb at 640 nm, which appears typically as a blue color, predicts the existence of approximate 40 conjugated carbons (10 DA monomers) in a polymer.^71^ Hence, it is presumed that blue-phased PDA obtained with CDP–DA is the result of at least 10 cross-linked conjugated DA monomers.

### Reversible thermochromism

**Fig. 11:**
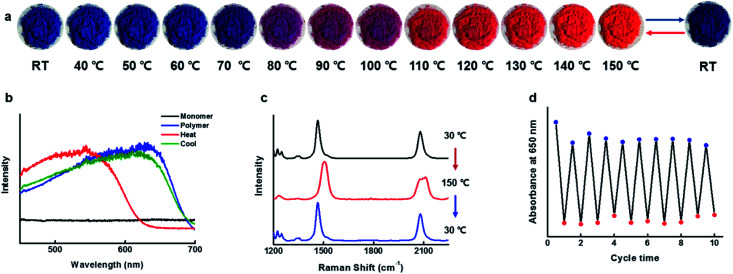
Reversible thermochromic property of CDP–DA in chloroform : methanol mixture at 1 : 2. (a) Digital images of thermochromic behaviour of polymerized CDP–PDA upon gradual heating from 30 °C to 150 °C. (b) Absorbance spectra of CDP–PDA showing thermochromic properties upon heating and cooling. (c) Raman spectra of CDP–PDA upon heating and cooling. (d) Plots of absorbance intensity at 650 nm as a function of thermal cycles (30 ↔ 150 °C).

The blue-phase PDAs known to possess tunable sensitivities to stimuli, as a result, produces a colorimetric response of brilliant blue-to-red color transition accompanied by optical changes. Since the blue phase PDA for CDP–DA nanotubes can be readily prepared by facile UV irradiation, subsequently, the temperature-dependent colorimetric response was investigated. The response of the CDP–DA nanotubes towards heat stimulus was evaluated in the solid phase by subjecting pristine material to gradual heating starting from 30 °C to 200 °C on a hot plate. The blue-phase PDA at 30 °C transformed into sharp red color at 110 °C through a transition bluish-purple phase during the heating temperature range between 80–100 °C, and reaches to the yellow phase when heated continuously further to 200 °C (Fig. S5 in ESI†). Very interestingly, the naked eye observations suggested that the thermochromic response of CDP–DA is fully reversible from a temperature of 150 °C (Fig. 11a). The reversible blue-to-red color transition during the thermal cycle at 30 ↔ 150 °C was assessed by UV-vis absorbance profiles. As seen in Fig. 11b, the maximal absorbance at 630 nm for the initial blue phase (30 °C) shifted to 544 nm upon heating to the red phase (150 °C). Upon cooling to 30 °C, a perfect reversible process with initial blue color regeneration for CDP–DA and absorption spectrum overlapping with the initial blue phase spectrum can be seen. The thermochromic reversibility of CDP–DA was further verified by the Raman spectroscopy. Raman spectra of CDP–DA recorded during consecutive blue-red-blue phase transition at respective 30-150-30 °C show complete reversibility (Fig. 11c). Furthermore, the repeatability of the reversible blue-red-blue color transition as a function of consecutive heating and cooling was feasible for more than a dozen of repetitive cycles without noticeable loss of absorption intensity (Fig. 11d). The morphological integrity of the CDP–PDA nanotubes during the thermal cycle was investigated with SEM, which showed retention of tubular features (Fig. S6 in ESI†).

### Hydrogen bonding

The robust intermolecular hydrogen bonding interactions originating from amide functionality is believed to act as a non-covalent ‘clamps’ to retain initial structural arrangement even if distortion among alkyl side chains on the PDA main chain upon heating at a higher temperature. This indicates that the hydrogen bonds are crucial to direct the self-assembly and the polymerizable orientation of DA chains in the nanotubular structures, and to induce thermochromatic reversibility *via* reversible conformation transformation mechanism. The presence of intermolecular hydrogen bonding interaction between the amides groups is validated by concentration-dependent ^1^H NMR measurements (Fig. S7a in ESI†). Upon increasing the CDP–DA concentration from 1 mM to 15 mM in chloroform-d, the two amide–NH protons of CDP head group display significant downfield shifts from the original chemical shift of 5.51, 5.77 and 6.17 ppm to 5.55, 5.90 and 6.32 ppm, respectively. Also, the amide–NH protons of CDP–PCDA linkage display a noticeable downfield shifts pattern upon increasing the concentration. Additionally, the hydrogen bonding was evidenced by the change in the amide N–H and carbonyl C=O stretching peak in FT-IR spectra when recorded in solid and solution state. The N–H stretching frequency of both cyclic and terminal amide group shifted to the lower frequency from 3348 and 3390 cm^−1^ in the solution state to 3301 and 3190 cm^−1^ in solid powder state (Fig. S7b in ESI†). Also, the amide carbonyl group is strongly affected by hydrogen bonding. A shift in the frequency for the amide carbonyl group was observed from 1678 cm^−1^ in the solution to 1685 and 1641 cm^−1^ in the powder state (Fig. S7c in ESI†). On the basis of these results, it can be concluded that the self-assembly and reversible thermochromism of CDP–DA is driven by intermolecular hydrogen bonding interactions.

## Conclusions

In summary, reversible high temperature thermochromic covalently-linked nanotubes were fabricated from amphiphilic CDP–PDA conjugate. This study demonstrates the concept of facile transformation of intrinsic supramolecular nanotubes of self-assembled linear CDP–DA molecules into rigid covalently cross-linked chromogenic nanotubes by UV induce topochemical polymerization. A linear structure, CDP–DA, comprising polar CDP head group linked to the diacetylene chain was synthesized and studied for supramolecular structures in different solvent systems. The CDP–DA-1 forms self-assembled architectures through cooperative non-covalent interactions involving intermolecular hydrogen bonding and π–π stacking character. Interestingly, different supramolecular architectures, including xerogel, dandelion-like microspheres consisting of plate-like and perfect tubular structures were observed during the solvent-polarity-tuned process, which were found to have different molecular arrangements. Solvent mixing is believed to exert a significant influence on the assemblies of the CDP–DA structures, which could tune the balance between the hydrogen bonding interactions of amides and π–π stacking of the DA template effectively and governs the final morphology of the CDP–DA. Detailed mechanistic studies using SEM, TEM, and XRD techniques illustrate that the self-assembly of CDP–DA forming into hollow nanotubular architecture is driven by the co-solvent polarity of chloroform/methanol mixture. At an optimized volume ratio of chloroform/methanol mixture (1 : 2 v/v), the bilayer lamellar CDP–DA-1 assemblies in nanosheet-like configuration undergo a scrolling mechanism to form single-/multi-wall supramolecular nanotubes. Eventually, the transition of supramolecular to rigid covalent polymer structure of CDP–DA nanotubes was achieved *via* UV-induced topochemical polymerization. As a result of polymerization forming extended conjugated PDA chain, CDP–DA displayed a brilliant blue color. The formation of polymeric CDP–PDA was confirmed by UV-vis and Raman spectral analysis. The SEM imaging of PDA tubes upon solvent exposure test evidences the high structural rigidity without any structural distortion. Very interestingly, the CDP–PDA displays a naked-eye detectable reversible thermochromic response. The thermochromic reversibility and repeatability of CDP–PDA-1 was demonstrated using UV absorption and Raman spectroscopic methods. The presence of intermolecular hydrogen bonding responsible for self-assembly and reversible thermochromic behavior was confirmed by ^1^H NMR and FT-IR studies.

The current study provides a facile avenue towards the creation of rigid covalently linked organic nanotubes of linear molecular hybrids from their supramolecular precursors. Also, signifies the role of co-solvent polarity interplay in regulating non-covalent interactions to tune the morphology. We hope that the temperature-dependent reversible color-change property of CDP–DA nanotubes has the potentials for application to the real-time temperature-sensing devices.

## Conflicts of interest

The authors declare no competing financial interest.

## Supplementary Material

RA-010-D0RA05656A-s001
