# Supplementary material for: Co-solvent polarity tuned thermochromic nanotubes of cyclic dipeptide–polydiacetylene supramolecular system
Source: RSC Adv. 2020 Sep 24;10(58):35389–96. doi: 10.1039/d0ra05656a (PMC9056892; doi:10.1039/d0ra05656a)
Supplement: RA-010-D0RA05656A-s001 [file RA-010-D0RA05656A-s001.pdf]

Electronic Supplementary Material (ESI) for RSC Advances.
This journal is © The Royal Society of Chemistry 2020

# Electronic Supplementary Information

## Co-solvent polarity tuned thermochromic nanotubes of cyclic dipeptide–polydiacetylene supramolecular system.

Mohammed Iqbal Khazi,[a] Chenikkayala Balachandra,[b] Geon Shin,[c] Gang-Hee Jang,[c] Thimmaiah Govindaraju[b,*] and Jong-Man Kim[a,c,*]

[a] Institute of Nano Science and Technology, Hanyang University, Seoul 04763, Korea
[b] Bioorganic Chemistry Laboratory, New Chemistry Unit and the School of Advanced Materials (SAMat), Jawaharlal Nehru Centre for Advanced Scientific Research, Jakkur P.O., Bengaluru, Karnataka 560064, India.
[c] Department of Chemical Engineering, Hanyang University, Seoul 04763, Korea.

*Corresponding authors:
E-mail address: tgraju@jncasr.ac.in (Thimmaiah Govindaraju) and jmk@hanyang.ac.kr (Jong-Man Kim)

| | **Contents** | **Page no.** |
|---|---|---|
| **1.** | **Experimental Section** | **2~3** |
| **2.** | **Synthesis of CDP-DA** | **4** |
| **3.** | **NMR and MALDI-TOF spectra for CDP-DA (Fig. S1~S2)** | **5~6** |
| **4.** | **Table for the morphology of CDP-DA in the different volume ratio of chloroform/ methanol mixture (Table S1)** | **7** |
| **5.** | **SEM images of CDP-DA for various solvents (Fig. S3)** | **8** |
| **6.** | **Powder X-ray diffraction pattern of CDP-DA (Fig. S4)** | **9** |
| **7.** | **Reversible thermochromic response of CDP-PDA (Fig. S5)** | **10** |
| **8.** | **SEM images of CDP-PDA nanotubes before and after heating (Fig. S6)** | **11** |
| **9.** | **$^1$H NMR and FT-IR spectra for hydrogen bonding effect of CDP-DA (Fig. S7)** | **12** |

## 1. Experimental section

### 1.1 Materials

L-Phenylalanine, 1-hydroxybenzotriazole hydrate, piperidine, triisopropylsilane and propylphosphonic anhydride (50% in ethyl acetate) were purchased from Sigma Aldrich (Korea). 10,12-Pentacosadiynoic acid (PCDA) was purchased from GFS Chemicals. Thionyl Chloride (1 mol/L in dichloromethane), 1-(3-dimethylaminopropyl)-3-ethylcarbodiimide hydrochloride, N,N-diisopropylethylamine, trifluoroacetic acid were purchased from TCI. Triethylamine was purchased from Daejung (Korea). All the other chemicals used were analytical grade reagents.

### 1.2 Instruments

SEM of nanotube morphology was conducted with a JEOL (JSM-6330F) FE-SEM. TEM investigations were carried out using a JEOL TEM-2100F microscope. The absorption spectra were recorded on a USB2000 miniature fiber-optic spectrometer (Ocean Optics). IR spectra were recorded on a Thermo Nicolet NEXUS 470 FTIR using an ATR accessory (Thermo Fisher Scientific, Inc.). The $^{1}H$ and $^{13}C$ NMR spectra were recorded on a Varian Unitylnova (300 and 600 MHz) spectrometer at 298 K in $CDCl_3$ and DMSO-$d_6$. Mass spectra were recorded on a AXIMA MALDI-TOF (SHIMADZU). XRD spectra were recorded with a minFlex600 (Rigaku, Germany). Raman spectra were recorded on a LabRAM HR Evolution Raman Spectrometer (Horiba Scientific, 785nm laser source).

## 1. Syntheses of CDP [Cyclo(L-Lys-L-Phe)]

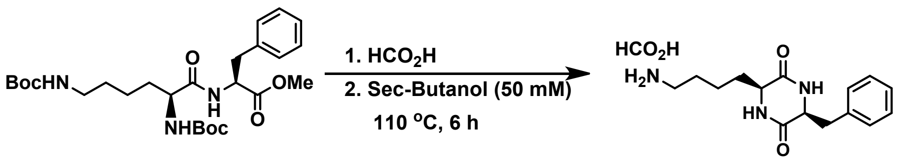


1

**Syntheses of (*3S,6S*)-3-(4-aminobutyl)-6- benzylpiperazine-2,5-dione) (1).** The cyclo (L-Lys-L-Phe) was synthesized by following the previously reported procedure.[1] The dipeptide εN-Boc, αN-Boc-Lys-Phe-OMe (5.1 g, 10.05 mmol) was subjected to Boc deprotection by treating with formic acid. The dipeptide was dissolved in formic acid (80 mL) and stirred at room temperature for 2 h. The reaction was monitored by thin layered chromatography (TLC). After completion of the reaction, all the volatiles were evaporated under reduced pressure. The obtained residue was co-evaporated with toluene (2 × 60 mL) and dried under vacuum for 30 min. Then the residue was re-dissolved in sec-butanol (195 mL, ~50 mM) and allowed to stir at 110 °C in an oil bath for 6 h. After 30 min, the formation of a white precipitate was observed, and a thick curdy white precipitate was formed with time. After 6 h, the reaction was stopped and brought to room temperature. Then the maximum amount of volatiles was evaporated using a rotary evaporator under reduced pressure and a thick white suspension was obtained. The thick white suspension was filtered under vacuum using a sintered funnel and was washed with dichloromethane. The white precipitate was dried under vacuum to obtain the pure product as a white solid (2.0 g, 67% yield) and was characterized by $^{1}$H &$^{13}$C NMR and HRMS analysis.

## 2. Synthesis of CDP-DA

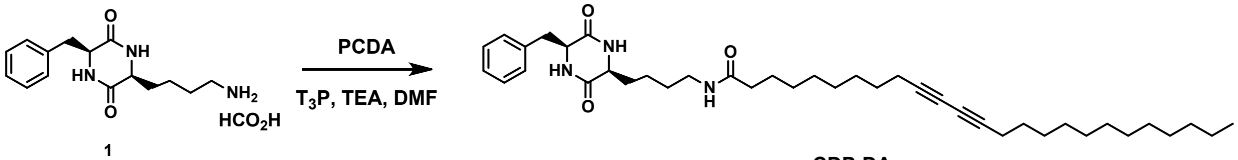


**N-(4-((2S,5S)-5-benzyl-3,6-dioxopiperazin-2-yl)butyl)pentacosa-10,12-diynamide (CDP-DA).** To a solution of cyclo(L-lys-L-phe) formic acid salt (250 mg, 0.311 mmol, 1 equiv), 10,12-pentacosadiynoic acid (350 mg, 0.373 mmol, 1.2 equiv) and triethylamine (0.55 mL, 1.245 mmol, 5 equiv) in 10 mL of DMF was added a solution of propylphosphonic anhydride (0.7 ml, 0.467 mmol, 1.5 equiv) at room temperature and stirred overnight at 60 °C. The reaction mixture was cooled at RT, and then solid was collected by filtration and washed with EA. The crude material was triturated /washed with ACN to afford title compound (220 mg, 45%). $^{1}$H NMR: (600 MHz, DMSO-d$_6$): δ 8.13 (d, *J* = 2.4 Hz, 1H), 8.03 (d, *J* = 2.4 Hz, 1H), 7.25 (t, *J* = 6 Hz, 2H), 7.20 (t, *J* = 6 Hz, 2H), 7.15 (d, *J* = 6 Hz, 1H), 4.18 (t, *J* = 6 Hz, 1H), 3.54 (t, J = 6 Hz, 1H), 3.14 (dd, 6 Hz, 12 Hz, 1H), 2.85-2.80 (m, 3H), 2.27 (t, *J* = 6 Hz, 4H), 2.01(t, *J*= 6 Hz, 2H), 1.49-1.41 (m, 6H), 1.32-1.24 (m, 24H), 1.10-1.01 (m, 4H), 0.85 (t, *J* = 6Hz, 3H), 0.77-0.71 (m, 2H), 0.65-0.59 (m, 2H); $^{13}$C NMR: (75 MHz, $CDCl_3$) 173.4, 167.4, 166.9, 135.2, 129.8, 129.0, 127.5, 65.2, 56.1, 54.7, 45.5, 39.8, 38.5, 37.6, 36.8, 32.7, 31.9, 30.2, 29.6, 29.4, 29.3, 29.2, 29.1, 29.0, 28.9, 28.8, 28.7, 28.3, 28.2, 25.7, 22.6, 21.3, 19.2, 16.0, 15.2, 14.1; IR (cm$^{-1}$) *v*max: 3302, 3190, 3057, 2919, 2848, 1685, 1640, 1541, 1453, 1338, 1253, 1218, 1191, 1102, 820, 744, 721, 697, 576, 480, 447; MS (MALDI-TOF, m/z): calcd. for $C_{40}H_{61}N_3O_3$ $[M+Na]^+$654.46, found 654.19

## References


1. C. Balachandra, T. Govindaraju, *J. Org. Chem.,* 2020, **85**, 1525–1536.

## 3. NMR and MALDI-TOF spectra for CDP-DA

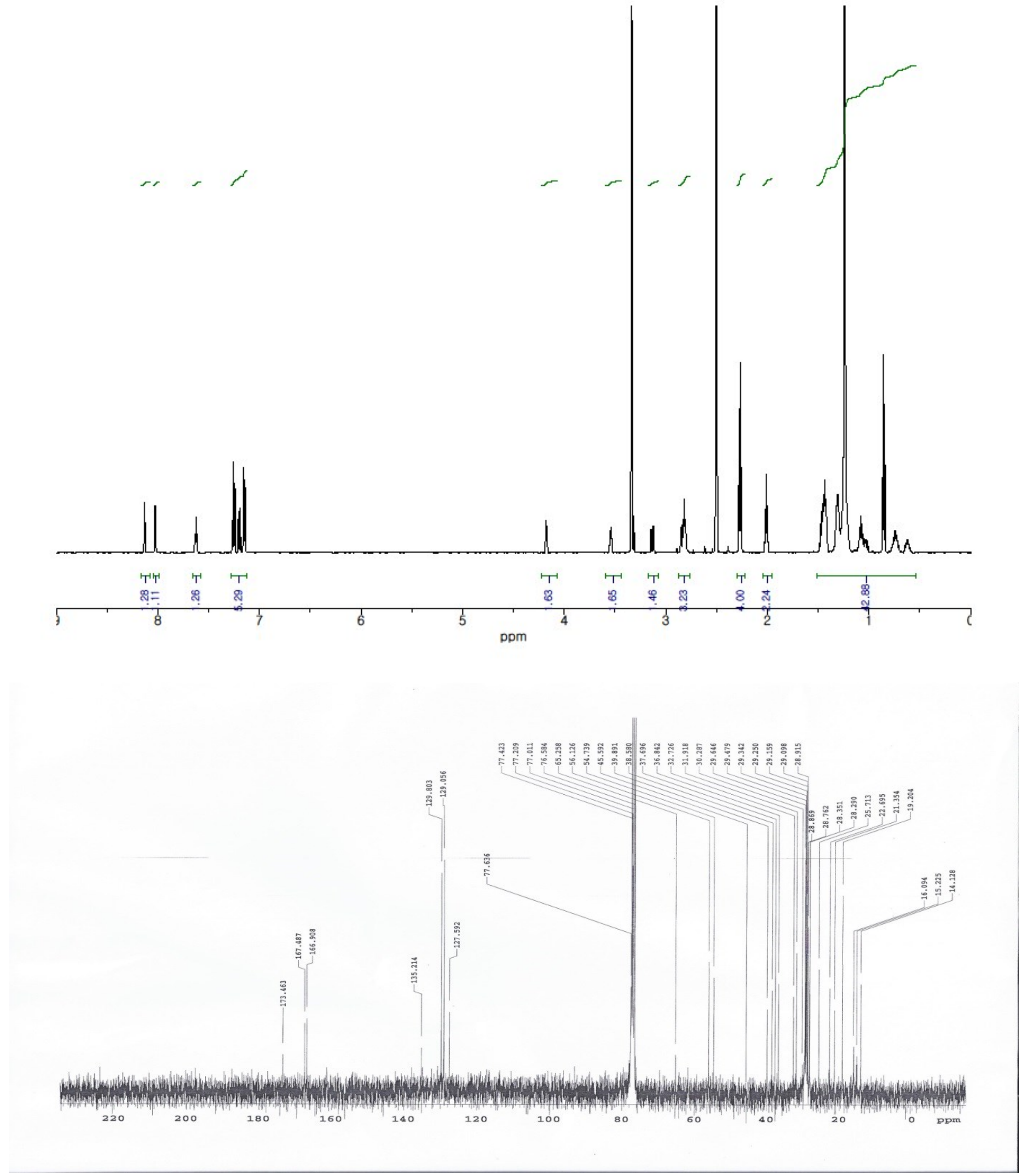


**Figure S1.** $^{1}H$ (top, 600 MHz, DMSO-$d_6$) and $^{13}C$ (bottom, 75 MHz, $CDCl_3$) NMR spectra of N-(4-((2S,5S)-5-benzyl-3,6-dioxopiperazin-2-yl)butyl)pentacosa-10,12-diynamide (CDP-DA).

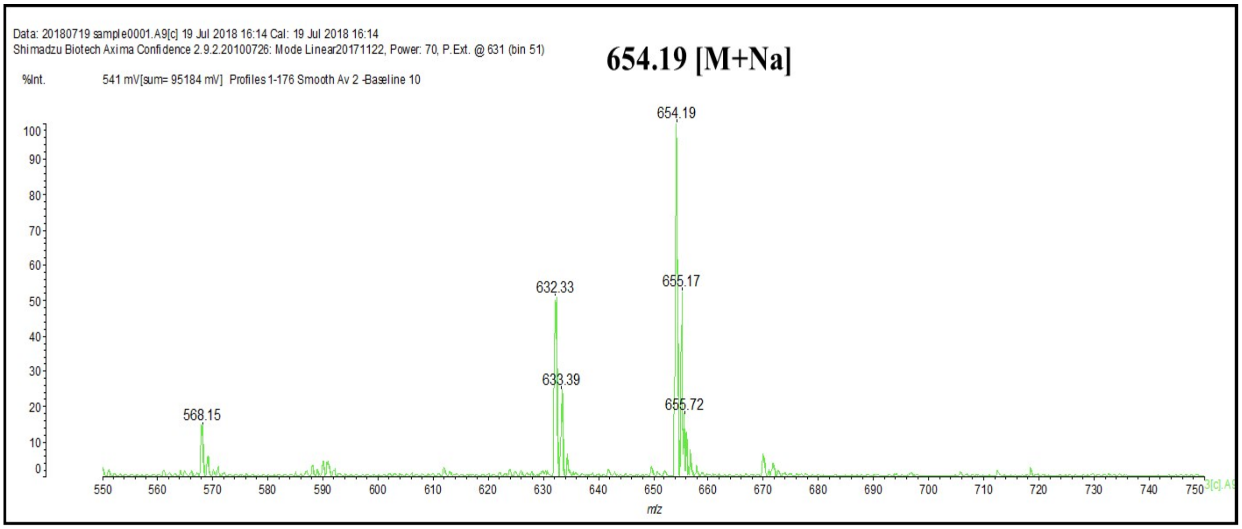


**Figure S2.** MALDI-TOF spectrum of N-(4-((2S,5S)-5-benzyl-3,6-dioxopiperazin-2-yl)butyl) pentacosa-10,12-diynamide(CDP-DA)**.** MS (MALDI-TOF, m/z): calcd. for $C_{56}H_{90}N_2O_6$ [M+Na] 654.46, found 654.19

**4. Table for morphologies of CDP-DA in the different volume ratio of chloroform/ methanol mixture**

| | Chloroform | Chloroform Methanol ( 3 : 1 ) | Chloroform Methanol ( 2 : 1 ) | Chloroform Methanol ( 1 : 1 ) | Chloroform Methanol ( 1 : 2 ) | Chloroform Methanol ( 1 : 3 ) | Methanol |
|---|---|---|---|---|---|---|---|
| **Phase** | Clear gel | Solution | Solution | Nanotube + Plate | Nanotube | Nanotube | Nanotube |
| **Polymerization** | X | X | X | O (Blue) | O (Blue) | O (Blue) | O (Blue) |

**Table S1**. Morphology of CDP-DA in the different volume ratio of chloroform/ methanol mixture

## 5. SEM images of CDP-DA in various solvents

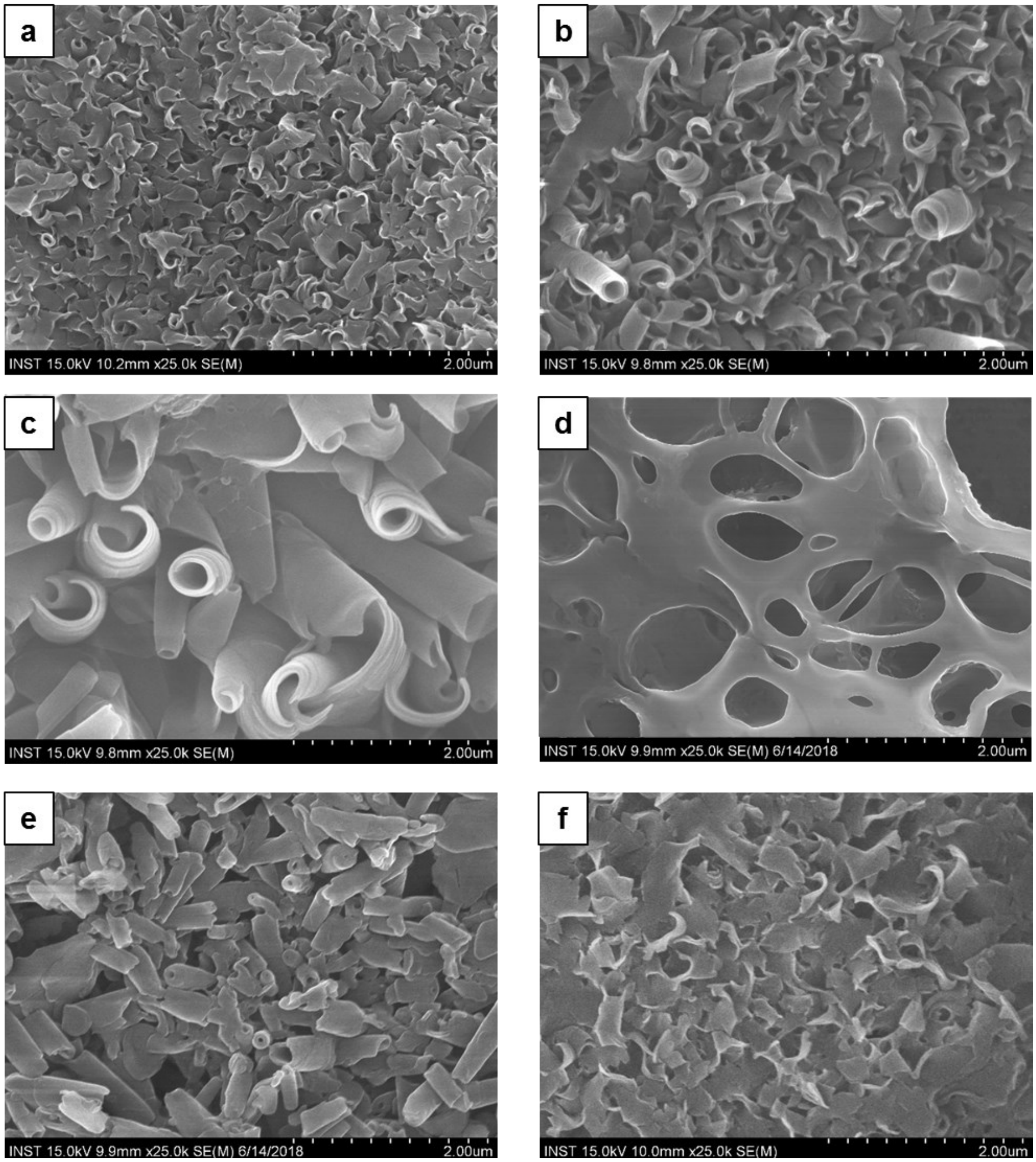


**Figure S3.** SEM images of CDP-DA in different solvent systems (a) tetrahydrofuran (b) ethyl acetate (c) isopropyl alcohol (d) dichloromethane (e) DMSO : Water (3:1 v/v) (f) acetone

## 6. Powder X-ray diffraction pattern of CDP-DA

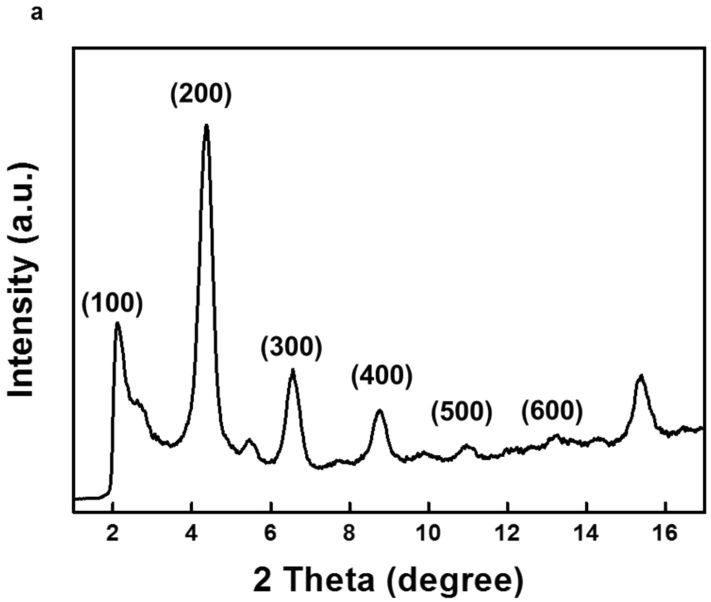


b

| 2Θ | d (Å) | |
|---|---|---|
| 2.12 | 41.6 | (100) |
| 4.36 | 20.8 | (200) |
| 5.46 | 16.2 | |
| 6.56 | 13.7 | (300) |
| 7.66 | 11.5 | |
| 8.76 | 10.8 | (400) |
| 10.82 | 8.2 | (500) |
| 13.2 | 7.0 | (600) |
| 15.4 | 5.5 | |

**Figure S4**. Powder X ray diffraction

**Figure S4.** Powder X-ray diffraction of CDP-DA (a) powder X-ray diffraction spectrum (b) Table of calculated 2θ theta values.

## 7. Thermochromic response of CDP-PDA

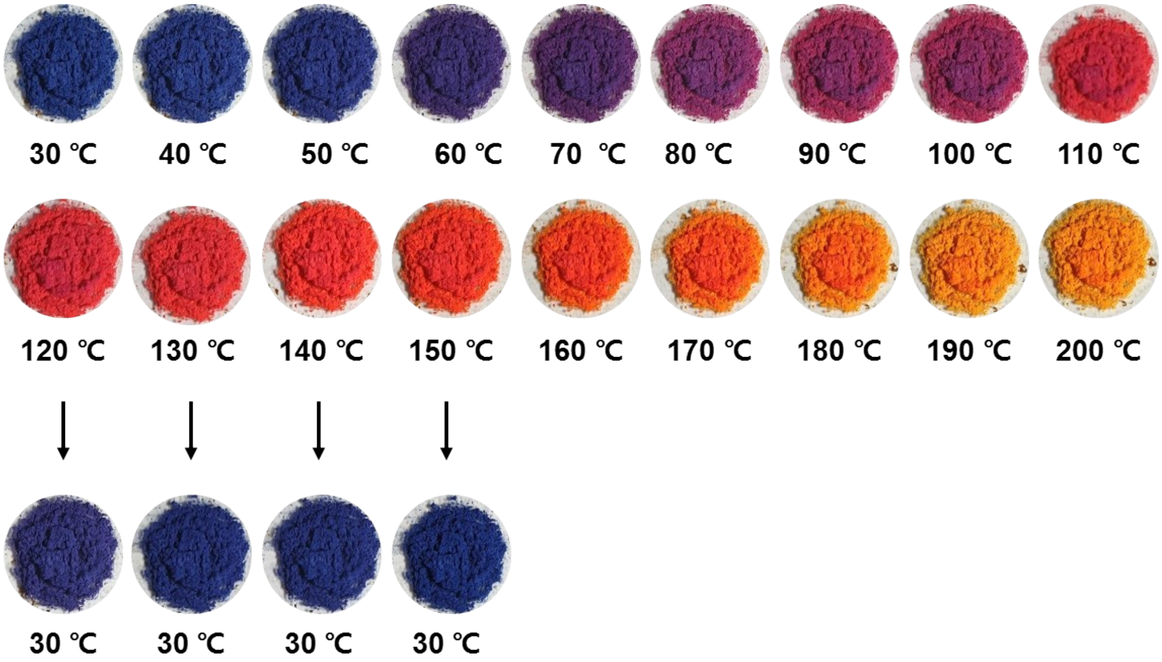


**Figure S5.** Thermochromic response of CDP-PDA 30 $^{o}$C to 200 $^{o}$C.

## 8. SEM images of CDP-PDA before and after heating

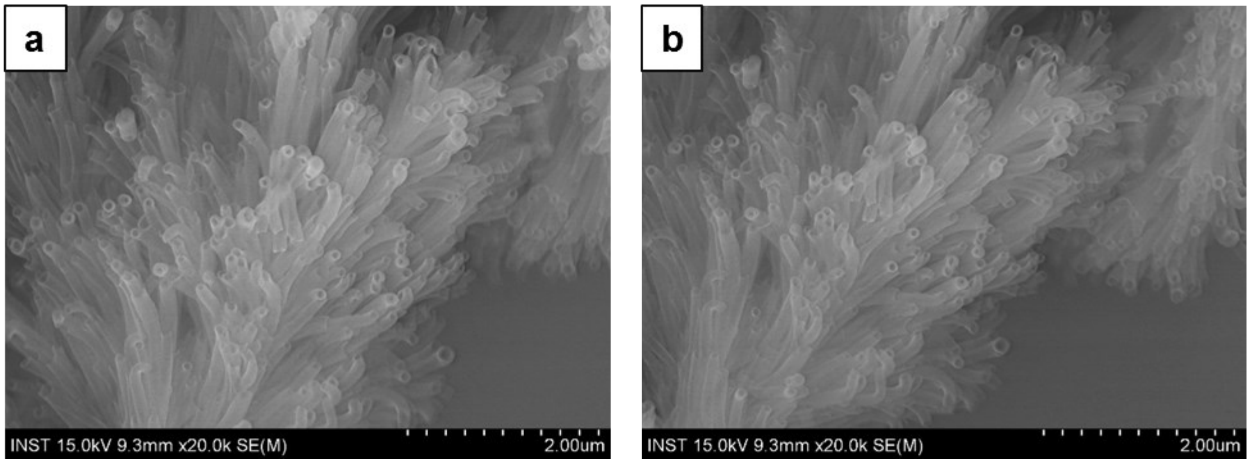


**Figure S6.** SEM images of CDP-PDA nanotubes (a) before and (b) after heating at 150 $^{o}$C for 5 minutes.

## 9. $^1$H NMR and FT-IR spectra for hydrogen bonding effect of CDP-DA

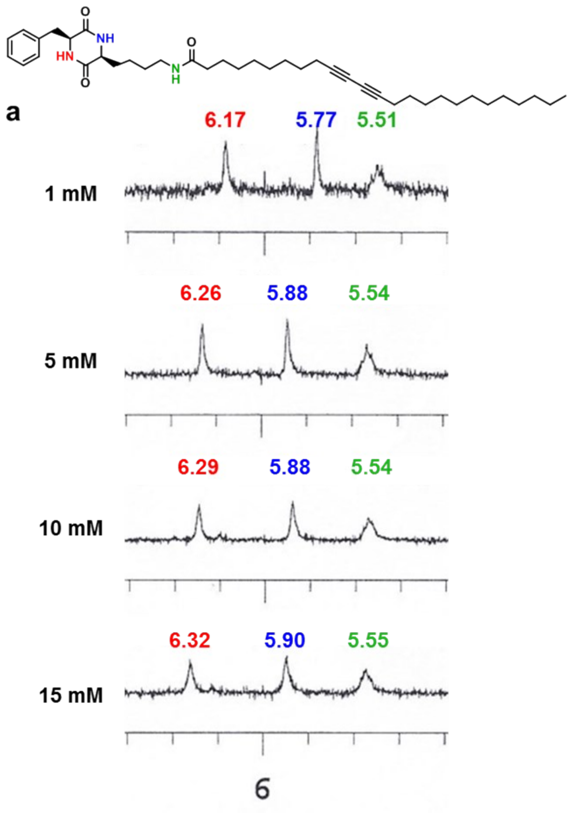


**Figure S7.** (a) $^1$H NMR ($CDCl_3$) peak shift of CDP-DA solution dependent on concentration. (b, c) FT-IR spectra of CDP-DA solution in chloroform (red) and powder (black).
